# Telecardiology Activities in Hospital and University Cardiology Facilities in Italy: Survey Study

**DOI:** 10.2196/73747

**Published:** 2025-12-05

**Authors:** Manuela Bocchino, Elvira Agazio, Cecilia Damiano, Giuseppe Di Lorenzo, Fabio De Paolis, Duilio Luca Bacocco, Fabrizio Ammirati, Alessio Nardini, Marco Silano

**Affiliations:** 1 Department of Cardiovascular, Endocrine-Metabolic Diseases and Aging, Istituto Superiore di Sanità, Roma, Italy; 2 Head of the Telemedicine UOS, General Management Staff, ASL Roma 3, Via Casal Bernocchi, 73Roma, 00125, Italy; 3 School of Specialization in Hygiene and Public Health, Sapienza University of Rome, Rome, Italy; 4 Office for General Affairs, Istituto Superiore di Sanità, Roma, Italy; 5 Emergency Department, Division of Cardiology, Azienda Sanitaria Locale Roma 3, Rome, Italy; 6 Ministero della Salute, Rome, Italy

**Keywords:** telemedicine, cardiology, survey, methodology, design

## Abstract

**Background:**

Telemedicine enables the provision of health services at a distance using information and communication technologies and includes different types of services: telemonitoring, remote control, virtual visit or televisit, telereferral, teleassistance, medical teleconsultation, health professionals’ teleconsultation, and telerehabilitation. Continuous monitoring, early care, and greater therapeutic adherence could be benefits of telemedicine in the management of cardiovascular diseases. There are not many studies in the literature investigating the use of telemedicine in cardiology in Italy.

**Objective:**

The aim of this study is to illustrate the results of a survey on telemedicine services in cardiology conducted by the Department of Cardiovascular, Endocrine-Metabolic Diseases and Aging of the Italian National Institute of Health.

**Methods:**

The Telehealth Quality of Care Tool (TQoCT) from the World Health Organization (WHO) was used as the model. A survey was disseminated by the National Association of Doctors and Hospital Cardiologists (ANMCO) from June 2024 to October 2024 through a link provided to hospital and university cardiology operative units identified through the 8th Census of Cardiological Structures in Italy. The facilities were contacted by email or telephone. The survey was built using Microsoft Forms and composed of 52 questions divided into 6 sections. The analysis was carried out for the whole national territory and by geographical area.

**Results:**

Of the 443 hospitals contacted, the response rate was 56.7% (251/443). Overall, 78.9% (198/251) of facilities reported telemedicine initiatives providing telemonitoring (128/198, 64.6%), telereferrals (104/198, 52.5%), medical teleconsultations (93/198, 47%), televisits (82/198, 41.4%), health professionals’ teleconsultations (64/198, 32.3%), and telerehabilitation (10/198, 5.1%). The most frequently followed cardiovascular conditions were heart failure, ischemic heart disease, and cardiac arrhythmias, especially atrial fibrillation. Of the facilities, 51% (101/198) used deliberations, procedures, protocols, or informed consent for their activities, and 46% (91/198) of the reported services were paid. Lack of dedicated staff, complexity in organizational terms, and lack of technological equipment in the structure were the principal obstacles for health professionals; lack of familiarity with technology was the principal obstacle for patients.

**Conclusions:**

There are still organizational and clinical limitations to resolve to make telemedicine in cardiology an integral part of medical practice. The true challenge of telecardiology is likely the integration of available technology with precise, concrete, and simplified organizational models. As a tool, technology is fundamental only if it is accessible and adequate. However, it must be integrated with new paths built according to the needs of the territory, patients, and health personnel. Such a survey could provide help for the future design and use of telemedicine services in cardiology in Italy.

## Introduction

### Background

According to the definition from the World Health Organization (WHO), telemedicine is the “provision of health services by all actors using information and communication technologies (ICT) for the exchange of valid information for diagnosis, treatment and prevention of diseases where distance is a critical factor, in the interest of improving the health of individuals and their communities” [1]. Telemedicine therefore allows remote health services to be delivered using ICT devices without patients having to go to a health care facility. Telemedicine includes different types of services, such as telemonitoring, remote control, virtual visit or televisit, telereferral, teleassistance, medical teleconsultation, health professionals’ teleconsultations, and telerehabilitation.

Regarding cardiology, telemedicine can be a useful tool for postdischarge care continuity, for example. Cardiology is a branch with a considerable number of chronic diseases, so continuous monitoring can help prevent and manage the worsening of a patient’s condition. Similarly, it allows remote data sharing, providing a comparison among health care professionals in a context that is not possible onsite.

In Italy, according to the mapping of experiences in telemedicine for the national territory drawn up in 2018 [2], telemonitoring and teleassistance represented 26% of the services, with 43% concerning cardiology specialists and 57% representing other specialists. Many regions and autonomous provinces took note of the 2020 national guidelines or interim indications for telemedicine care services [3,4] in different documents that proposed and implemented local solutions. In the field of cardiology, the “National Consensus Document on telemedicine for cardiovascular diseases: indications for tele rehabilitation and telemonitoring” [5] was recently published. Although telecardiology proved beneficial in terms of care, comfort, and patient access, it still has limitations, both logistical and medical, to becoming an integral part of medical practice in the future [6].

In 2021, the Italian Association of Arrhythmology and Cardiostimulation (AIAC) released a survey entitled “Telecardiology and the use of remote monitoring of implantable Devices in Italy in light of the COVID-19 period.” That survey aimed mainly to investigate the spread of remote monitoring of cardiac implantable electronic devices (CIEDs) in Italy, in particular following the period of the health emergency [7]. Subsequently, in October 2023, the Italian National Institute of Health (ISS) collaborated on the “First national survey on telemedicine in private outpatient care” with Luiss University, the Health Wellness and Resilience Observatory of the Bruno Visentini Foundation, and the integrative health fund Fasdac [8]. This survey concerned the use of telemedicine within private outpatient facilities. Recently, the 8th Census of Cardiological Structures in Italy, conducted by the National Association of Doctors and Hospital Cardiologists (ANMCO), reported some data about the spread of telemedicine in cardiology in Italy, but it only covered the frequency of use of televisit services, telemonitoring, teleconsultations, and telereferrals. The census was conducted in 2022 and published in the beginning of 2024 [9]. At the international level, the literature covers surveys on perceptions of health professionals about telemedicine during the COVID-19 pandemic [10] and the acceptance of telemedicine in cardiology by specialists and general practitioners [11]. Currently, there are no dedicated surveys reported in the literature that investigated telemedicine services and their characteristics in cardiology in Italy.

### Aim of the Study

This study reports the results of the survey on telecardiology in Italy, which was conceived, designed, and conducted by the Department of Cardiovascular, Endocrine-Metabolic Diseases and Aging of the ISS. The survey was addressed to hospital and university cardiology operative units (OUs) in the national territory—both those listed by ANMCO and those found using an online search.

The aim was to describe the current state of telecardiology services in Italy: Being aware of real-world data on the territory is essential to be able to understand the strengths and weaknesses of a service as well as to improve it.

## Methods

This report was conducted in adherence with CHERRIES (Checklist for Reporting Results of Internet E-Surveys) [12]. The Telehealth Quality of Care Tool (TQoCT) [13] was also consulted. Based on previous recommendations and scoreboards, the WHO developed a tool that serves both as a guide and as a self-assessment to improve the quality of telemedicine services, which is useful at the local, regional, and national levels.

### Recruitment

The respondents were selected on the basis of the 8th Census of Cardiological Structures in Italy conducted by ANMCO [9] in 2022. We also performed an online research of the institutional sites of the Italian regional health service for a better understanding of the real operational structures.

The questionnaire was disseminated in 3 levels of intervention: email with a cover letter sent to the health departments of the regions and autonomous provinces, email with a cover letter addressed to the Directors of the hospital and university cardiology OUs, and phone contact with the Directors of the cardiology hospital and university OUs.

An ISS email account was specifically created for the survey (surveytelecardiologia@iss.it) as well as the cover letters.

The institutional emails of the Directors of the OUs were used as contact details, unless they had explicitly requested to send them to their personal email address; the publicly available phone numbers of the OUs or numbers voluntarily provided by Directors themselves were used for telephone contact. The survey started on June 14, 2024, and ended on October 18, 2024.

To ensure widespread territorial coverage, several scientific societies were involved in survey dissemination, by sending emails with a cover letter to societies’ Presidents. In particular, the Italian Society of Cardiology; the AIAC, especially the Women & Arrhythmology Area; the Latium section of the Italian Society of Telemedicine; and the Italian Society of Digital Medicine actively contributed to the study.

A Microsoft Forms template was designed to ensure that the IP addresses of the computers used to complete the survey were not recorded. For each participating facility, the Microsoft Forms application created a unique ID and collected information on start time and completion time of data entry. The data controller was ISS as well as the participating centers.

### The Questionnaire

The survey, digitally composed using Microsoft Forms, was based on a questionnaire of 52 questions divided into 6 sections: (1) facility information (6 questions); (2) presence of telemedicine projects (1 question); (3) type of telemedicine services (27 questions) defined according to the 2020 State-Regions Agreement [4]; (4) governance, safety, and accessibility (12 questions); (5) volumes and patient centrality (4 questions); and (6) obstacles to service development and implementation (2 questions).

Of the 52 total questions, 18 were multiple choice, and 11 included a text description field, 4 of which were dedicated to the description of the structure. In addition, 16 required a single answer, while 6 required date entries and 1 question required numerical data. The complete questionnaire is available in Multimedia Appendix 1.

### Statistical Analysis

Statistical elaboration of the collected data was performed using R software (version 4.0.3) both on a national level and by geographical area. In particular, regions and autonomous provinces were divided into the 3 macro-areas of “north” (Aosta Valley, Piedmont, Liguria, Lombardy, Trentino-Alto Adige, Veneto, Friuli-Venezia Giulia, and Emilia-Romagna), “center” (Tuscany, Marche, Umbria, Latium, and Abruzzo), and “south and islands” (Molise, Campania, Puglia, Basilicata, Calabria, Sicily, and Sardinia). Quantitative variables are expressed as n (%).

### Ethical Considerations

The survey was submitted to the National Ethics Committee for Trials of Public Research Institutions and other public bodies with a national character (National Ethics Committee), which approved the study on May 17, 2024 (protocol number 0021567). The activity did not collect personal data; in any case, the study information and consent to participate in the study were an integral part of the form accessed through the link shared to participate in the survey itself.

## Results

### Facility Information

Of 443 facilities contacted, 251 responded, for a response rate of 56.7%. Table 1 shows the distribution of contacted and responding centers, along with their response rates, reported by region or autonomous province, and geographical areas.

**Table 1.** Contacted centers and responding centers reported by region or autonomous province and geographical area.

Location	Contacted centers (n=443), n (%)	Responding centers (n=251), n (%)^a^
Region		
Abruzzo	13 (2.9)	0 (0)
Basilicata	4 (0.9)	1 (25)
Calabria	14 (3.2)	7 (50)
Campania	46 (10.4)	12 (26.1)
Emilia-Romagna	29 (6.5)	19 (65.5)
Friuli-Venezia Giulia	8 (1.8)	5 (62.5)
Latium	47 (10.6)	36 (76.6)
Liguria	19 (4.3)	13 (68.4)
Lombardy	60 (13.5)	36 (60.0)
Marche	13 (2.9)	5 (38.5)
Molise	4 (0.9)	1 (25)
Piedmont	31 (7)	25 (80.6)
Puglia	29 (6.5)	14 (48.3)
Sardinia	10 (2.3)	9 (90)
Sicily	41 (9.3)	19 (46.3)
Tuscany	30 (6.8)	22 (73.3)
Trentino-Alto Adige	5 (1.1)	3 (60)
Umbria	8 (1.8)	5 (62.5)
Aosta Valley	1 (0.2)	1 (100)
Veneto	31 (7)	18 (58.1)
Geographical area		
North	184 (41.5)	120 (65.2)
Center	98 (22.1)	68 (69.4)
South and islands	161 (36.3)	63 (39.1)

a Percentage of those who were contacted in that region.

The mean time to complete the survey was 12 (SD 5) minutes. About one-half (123/251, 49%) of the responding facilities were part of hospitals directly managed by local health companies, followed by hospital and university companies (Table S1 in Multimedia Appendix 2).

### Presence of Telemedicine Projects

In total, 198 facilities (198/251, 78.9%) reported telemedicine initiatives; details by geographical areas are shown in Table 2.

**Table 2.** Facilities using telemedicine by geographical area.

Use of telemedicine	North (n=120), n (%)	Center (n=68), n (%)	South and islands (n=63), n (%)	Total (n=251), n (%)
Yes	101 (84.2)	53 (77.9)	44 (69.8)	198 (78.9)
No	19 (15.8)	15 (22.1)	19 (30.2)	53 (21.1)

### Type of Telemedicine Services

Since the same facility could perform more than just one telecardiology service, respondents were able to answer this question with multiple options. We found that the majority of facilities (128/198, 64.6%) provided telemonitoring services, followed by telereferrals (104/198, 52.5%), medical teleconsultations (93/198, 47%), televisits (82/198, 41.4%), health professionals’ teleconsultations (64/198, 32.3%), and telerehabilitation (10/198, 5.1%). Table 3 shows the breakdown by geographical area.

**Table 3.** Telemedicine services provided by facilities using telemedicine, reported by geographical area.

Telemedicine service	North (n=101), n (%)	Center (n=53), n (%)	South and islands (n=44), n (%)	Total (n=198, n (%)
Televisit	50 (49.5)	24 (45.3)	8 (18.2)	82 (41.4)
Medical teleconsultation	52 (51.5)	28 (52.8)	13 (29.6)	93 (47)
Health professionals’ teleconsultation	31 (30.7)	21 (39.6)	12 (27.3)	64 (32.3)
Telemonitoring	73 (72.3)	34 (64.2)	21 (47.7)	128 (64.6)
Telereferral	54 (53.5)	27 (50.9)	23 (52.3)	104 (52.5)
Telerehabilitation	8 (7.9)	2 (3.8)	0 (0)	10 (5.1)

Table 4 reports the cardiovascular diseases that were most commonly followed through different telemedicine services: patients with heart failure, carriers of CIEDs, or patients who needed therapeutic plans related to cardiovascular diseases.

**Table 4.** Cardiovascular diseases for which a national telemedicine service was provided in facilities using telemedicine (n=198).

Disease	Televisit (n=82), n (%)	Medical teleconsultation (n=93), n (%)	Health professionals’ teleconsultation (n=64), n (%)	Telemonitoring (n=128), n (%)	Telerehabilitation (n=10), n (%)
Ischemic heart disease	19 (23.2)	51 (54.8)	16 (25)	6 (4.7)	5 (50)
Congenital heart disease	1 (1.2)	16 (17.2)	1 (1.6)	—^a^	1 (10)
Valvular heart disease	3 (3.7)	32 (34.4)	6 (9.4)	1 (0.8)	3 (30)
Remote control of CIED^b^	55 (67.1)	36 (38.7)	37 (57.8)	90 (70.3)	—
Atrial fibrillation	20 (24.4)	39 (41.9)	16 (25)	46 (35.9)	—
Arterial hypertension	8 (9.8)	21 (22.6)	6 (9.4)	5 (3.9)	—
Rare diseases in cardiology	8 (9.8)	14 (15.1)	3 (4.7)	3 (2.3)	1 (10)
Arrhythmic disorders	15 (18.3)	35 (37.6)	17 (26.6)	41 (32)	2 (20)
Treatment plans for cardiovascular diseases	40 (48.8)	—	—	—	—
Noncardiological diseases	—	12 (12.9)	4 (6.2)	—	—
Heart failure	64 (78)	64 (68.8)	40 (62.5)	69 (53.9)	8 (80)
Syncope	8 (9.8)	26 (28)	14 (21.9)	41 (32)	—

a Not applicable.

b CIED: cardiac implantable electronic device.

A televisit was often used to follow up heart failure, for the remote control of CIEDs, and for the compilation of therapeutic plans of the drugs monitored by the Italian Agency of the Drug. Medical teleconsultations and health professionals’ teleconsultations were associated mainly with heart failure. Telemonitoring was mainly used with patients with CIEDs and to a lesser extent with patients using wearable devices, or at least not implantable devices, who had heart failure and atrial fibrillation. Finally, heart failure and ischemic heart disease were the pathologies for which telerehabilitation was used most often.

Telereferral of cardiological instrumental examinations was primarily used for the remote interpretation of electrocardiograms (ECGs), particularly within hospital-to-territory, intrahospital, and emergency network settings. Further details are shown in Table 5.

**Table 5.** Services that involved telereferrals at the national level and by geographical area.

Service	North (n=54), n (%)	Center (n=27), n (%)	South and islands (n=23), n (%)	Total (n=104), n (%)
Remote control of CIED^a^	34 (63)	14 (51.8)	8 (34.8)	56 (53.8)
Emergency ECG^b^ (network 118)	38 (70.4)	9 (33.3)	15 (65.2)	62 (59.6)
Intrahospital ECG	41 (75.9)	19 (70.4)	21 (91.3)	81 (77.9)
Hospital-territory ECG	42 (77.8)	13 (48.1)	7 (30.4)	62 (59.6)
Echocardiogram	5 (9.3)	1 (3.7)	3 (13)	9 (8.7)
ECG Holter monitoring for 24 hours	9 (16.7)	8 (29.6)	5 (21.7)	22 (21.8)
ECG monitoring through other devices	7 (13)	3 (11.1)	3 (13)	13 (12.5)
Outpatient monitoring of blood pressure	1 (1.8)	6 (22.2)	3 (13)	10 (9.6)

a CIED: cardiac implantable electronic device.

b ECG: electrocardiogram.

For some telemedicine services, additional elements were investigated (ie, the facilities [hubs and spoke] in which the medical teleconsultation was carried out and number and skills of the health personnel involved in teleconsultation and telemonitoring).

Medical teleconsultations were carried out mainly with other regional hospitals and with general practitioners or free choice pediatricians, followed by the OUs of the same hospital (Table 6). There was still little interaction between regions, although this was present at greater proportions in the regions of central Italy; in the south, at a territorial level, interactions with territorial cardiology prevailed rather than with general medicine.

**Table 6.** Facilities in which medical teleconsultation was carried out at the national level and by geographical area.

Type of structure	North (n=52), n (%)	Center (n=28), n (%)	South and islands (n=13), n (%)	Total (n=93), n (%)
National hospitals or referral centers for pathology	7 (14)	5 (18)	1 (8)	13 (14)
Regional hospitals	27 (52)	13 (46)	6 (46)	46 (50)
Territorial cardiology clinics	7 (14)	2 (7)	6 (46)	15 (16)
Specialist noncardiological territorial	4 (8)	1 (4)	0 (0)	5 (5)
General practitioners or free choice pediatricians	27 (52)	5 (18)	3 (23)	35 (38)
Private or private/contracted facilities	5 (10)	1 (4)	3 (23)	9 (10)
OUs^a^ of the same hospital	9 (17)	15 (54)	9 (69)	33 (36)

a OU: operative unit.

Hub centers accounted for 30% (28/93), spoke centers accounted for 40% (37/93), and the remaining worked as both hub and spoke centers (details by geographical area are shown in Table S4 in Multimedia Appendix 2).

The health professionals’ teleconsultation service was primarily conducted by nurses, followed by cardiocirculatory pathophysiology technicians and, at lower percentages, doctors, physiotherapists, and bioengineers. Details can be found in Table 7.

**Table 7.** Staff providing health professionals’ teleconsultation services at the national level and by geographical area.

Professional role	North (n=31), n (%)	Center (n=21), n (%)	South and islands (n=12), n (%)	Total (n=64), n (%)
Bioengineers	0 (0)	2 (10)	2 (17)	4 (6)
Cardiologists	0 (0)	0 (0)	2 (17)	2 (3)
Physiotherapists	1 (3)	0 (0)	1 (8)	2 (3)
Nurses	26 (84)	13 (62)	7 (58)	46 (72)
Doctors	4 (13)	4 (19)	3 (25)	11 (17)
Doctors-radiology technicians	1 (3)	0 (0)	0 (0)	1 (2)
Cardiocirculatory pathophysiology technicians	8 (26)	6 (29)	2 (17)	16 (25)

Telemonitoring was primarily managed by doctors and, to a lesser extent, by nurses and cardiocirculatory pathophysiology technicians (Table S3 in Multimedia Appendix 2). In particular, nursing staff performed more telemonitoring services in the north than in the center and south; cardiocirculatory pathophysiology technicians were more involved in the north and center, less in the south (19%, 4/21).

### Governance, Safety, and Accessibility

Only 46% (91/198) of the centers were subjected to reimbursement. The reimbursement mainly concerned televisits and remote control (ie, remote control of the CIED) and, to a lesser extent, telemonitoring, teleconsultations, and other services (Table 8).

**Table 8.** Presence of reimbursement for telemedicine services and service types at the national level and by geographical area.

Reimbursement characteristics	North (n=101), n (%)	Center (n=53), n (%)	South and islands (n=44), n (%)	Total (n=198), n (%)
Charges				
Yes	56 (55.4)	27 (50.9)	8 (18.2)	91 (46)
No	45 (44.6)	26 (49.1)	36 (81.8)	107 (54)
Services subject to charges				
Renewal of treatment plans	0 (0)	0 (0)	1 (12.5)	1 (1.1)
Health professionals’ teleconsultations	5 (10.9)	2 (7.4)	0 (0)	7 (7.7)
Medical teleconsultations	8 (14.3)	4 (57.1)	0 (0)	12 (13.2)
Remote control (CIED^a^)	32 (57.1)	10 (37)	5 (62.5)	47 (51.6)
Telemonitoring	16 (28.6)	2 (7.4)	2 (25)	20 (22)
Telerehabilitation	4 (7.1)	0 (0)	0 (0)	4 (4.4)
Televisits	31 (55.3)	23 (85.2)	5 (62.5)	59 (64.8)
I don’t know	1 (1.8)	1 (3.7)	0 (0)	2 (2.2)

a CIED: cardiac implantable electronic device.

Considering the facilities that provided telemedicine services, in 51% of cases (101/198), company deliberations, specific procedures, operational protocols, or informed consent are used. In 43% of the cases (86/198), the structure did not prepare any of the aforementioned documents for the personnel addresses (Table 9). For more detailed information on the type of documents adopted, please consult Table S4 in Multimedia Appendix 2.

**Table 9.** In the facilities using telemedicine, presence of deliberations, procedures, protocols, or informed consent at the national level and by geographical area.

Presence of the documents	North (n=101), n (%)	Center (n=53), n (%)	South and islands (n=44), n (%)	Total (n=198), n (%)
Yes	57 (57.4)	26 (49.1)	17 (38.6)	101 (51)
No	40 (39.6)	22 (41.5)	24 (54.6)	86 (43.4)
I don’t know	2 (2)	5 (9.4)	0 (0)	7 (3.6)
Other	1 (1)	0 (0)	3 (6.8)	4 (2)

From a technological point of view, 52% (103/198) of the centers used devices (47/101, 46.5% in the north; 27/53, 51% in the center; and 28/44, 64% in the south), including medical devices in 86% (89/198; 88/101, 87.1% in the north; 47/53, 89% in the center; and 36/44, 82% in the south) of centers. Table 10 shows the device types used; for further details on device types, refer to Table S5 in Multimedia Appendix 2.

**Table 10.** Devices used in telemedicine facilities nationally and by geographical area.

Device	North (n=101), n (%)	Center (n=53), n (%)	South and islands (n=44), n (%)	Total (n=198), n (%)
Implantable device^a^	28 (27.7)	15 (28.3)	13 (29.5)	56 (28.3)
Device for monitoring basic clinical parameters^b^	59 (58.4)	35 (66)	44 (100)	138 (69.7)
ECG^c^	18 (17.8)	12 (22.6)	18 (41)	48 (24.2)
Ultrasound	0 (0)	1 (1.9)	1 (2.3)	2 (1)
Event recorder	0 (0)	1 (1.9)	0 (0)	1 (0.5)
Spirometer	3 (2.3)	1 (1.9)	0 (0)	4 (2.0)
Other	1 (1.0)	0 (0)	0 (0)	1 (0.5)

a Hemodynamic monitoring devices, loop recorder, pacemaker also biventricular, and defibrillators also biventricular.

b Physical activity, sleep, weight, heart rate, blood pressure, glycemia, body composition and hydration status of the body, pulse oximetry, temperature, and respirator frequency.

c ECG: electrocardiogram.

An interesting aspect concerned the health care workers’ access to telemedicine services. Only 56 of the 198 respondents (28.3%) had a telemedicine center with dedicated staff (33/101, 32.7% in the north; 13/53, 24% in the center; and 10/44, 23% in the south) in the facility. The facility or OU provided training for health personnel to start telemedicine services in 56% of cases (111/198; 64/101, 63.4% in the north; 24/53, 45% in the center; and 24/44, 54% in the south). Only 39 of the 198 facilities (19.7%) were able to collect data.

### Volume and Patient Centrality

The number of patients treated by most facilities ranged from 100 to 500 annually; details are given in Table 11.

**Table 11.** Patients followed yearly through telemedicine at the national level and by geographical area.

Number of patients treated using telemedicine	North (n=101), n (%)	Center (n=53), n (%)	South and islands (n=44), n (%)	Total (n=198), n (%)
0‐100	13 (12.9)	13 (24.6)	11 (25)	37 (18.7)
101‐500	62 (61.4)	33 (62.2)	29 (65.9)	124 (62.6)
>500	26 (25.7)	7 (13.2)	4 (9.1)	37 (18.7)

The majority of facilities reported adequate information services provided to patients, and communication was mostly personalized at the start of the patients’ treatment (83/198, 41.9%); information services included informative brochures, face-to-face meetings, or digital material. In 25.3% of cases (50/198), none of these instruments were provided. The detail, at the national level and by geographical area, is shown in Table 12.

**Table 12.** Information tools for patients and their caregivers on telemedicine services and the use of necessary technology at the national level and by geographical area.

Information tools	North (n=101), n (%)	Center (n=53), n (%)	South and islands (n=44), n (%)	Total (n=198), n (%)
In customized form at the beginning of the treatment pathway	52 (51.7)	23 (44.1)	9 (20.6)	83 (41.9)
Informative brochures	24 (23.3)	9 (16.2)	6 (12.7)	37 (18.7)
Events in attendance	11 (10.8)	2 (1.9)	3 (6.3)	15 (7.6)
Digital material on the website	3 (3.3)	2 (1.9)	0 (0)	5 (2.5)
None of these	20 (19.8)	13 (23.5)	17 (38.1)	50 (25.3)

In only 20.2% (40/198) of the cases, a patient satisfaction assessment was carried out through questionnaires (27/40, 68%) or interviews (13/40, 32%).

### Obstacles to Service Development and Implementation

The obstacles to the development of telemedicine are reported in Table 13. The most reported problems were a lack of dedicated personnel, organizational complexity, and a lack of technological endowments available in the structure. Regulatory problems relating to the lack of clear and simple rules for all and technological barriers, such as poor connectivity and poor digital information of staff, were also present. Data about obstacles were similar between facilities not providing telemedicine services and those providing them.

**Table 13.** Obstacles encountered in the adoption of telemedicine services at the national level and by geographical area.

Obstacles encountered	Geographical area			Use of telemedicine	
	North (n=101), n (%)	Center (n=53), n (%)	South and islands (n=44), n (%)	No (n=53), n (%)	Yes (n=198), n (%)
No obstacles	5 (4.2)	4 (5.9)	2 (3.2)	1 (1.8)	10 (5)
Lack of legislation	26 (21.7)	15 (22.1)	17 (27.0)	7 (13.2)	51 (25.7)
Complexity in organizational terms	63 (52.5)	37 (54.4)	35 (55.6)	23 (43.4)	112 (56.6)
Complexity in the application of GDPR^a^	15 (12.5)	5 (7.4)	9 (14.3)	2 (3.8)	27 (13.6)
Complexity in the application of specific legislation	16 (13.3)	4 (5.9)	8 (12.7)	7 (13.2)	21 (10.6)
Mistrust and lack of confidence in telemedicine	7 (5.8)	3 (4.4)	6 (9.5)	2 (3.8)	14 (7.1)
Data fragmentation and noninteroperability of systems	26 (21.7)	17 (25)	17 (27)	6 (11.3)	54 (27.3)
Lack of technological equipment in the structure	44 (36.7)	34 (50)	24 (38.1)	23 (43.4)	79 (39.9)
Lack of adequate infrastructure and Internet connectivity	28 (23.3)	17 (25)	18 (28.6)	8 (15.1)	55 (27.8)
Lack of dedicated staff	64 (53.3)	38 (55.9)	47 (74.6)	30 (56.6)	119 (60.1)
Cost in economic terms	21 (17.5)	7 (10.3)	11 (17.5)	8 (15.1)	31 (15.6)
Internal procedures inappropriate to the theme	19 (15.8)	6 (8.8)	11 (17.5)	5 (9.4)	31 (15.6)
Poor digital training of the staff	12 (10)	12 (17.6)	10 (15.9)	5 (9.4)	29 (14.6)
Lack of staff willingness or cooperation	6 (5)	7 (10.3)	6 (9.5)	4 (7.5)	15 (7.6)

a GDPR: General Data Protection Regulation.

**Figure 1.**
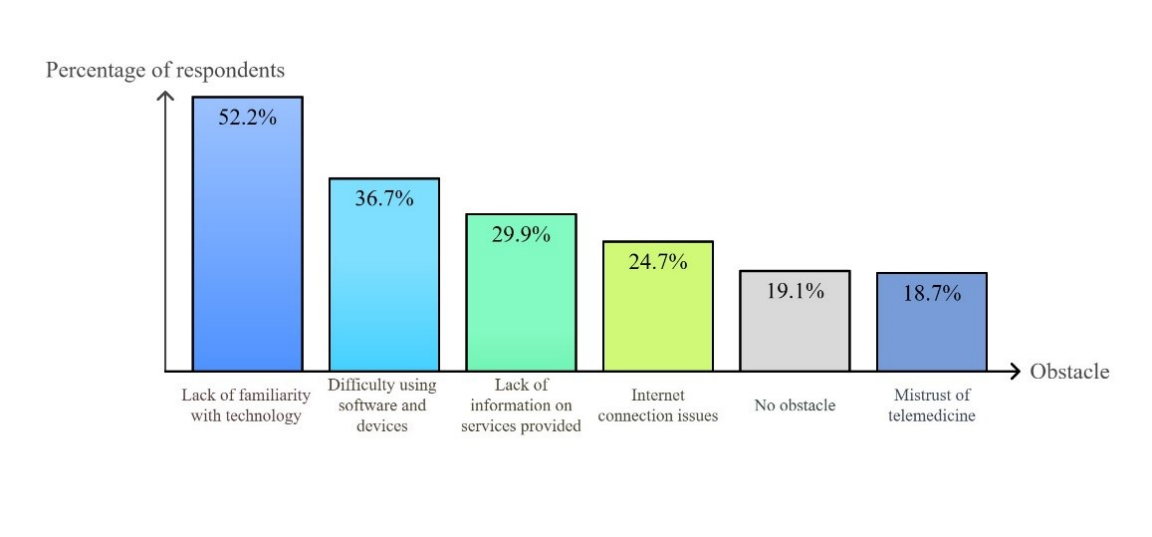
Obstacles to the adoption of telemedicine tools by patients at the national level.

The obstacles to the development of telemedicine encountered by patients included a lack of familiarity with technology, followed by difficulty with using software and devices, lack of information, and poor home internet connection. Distrust toward these new services concerned only 35 of the 198 centers (17.7%). Details are shown in Figures1  2.

**Figure 2.**
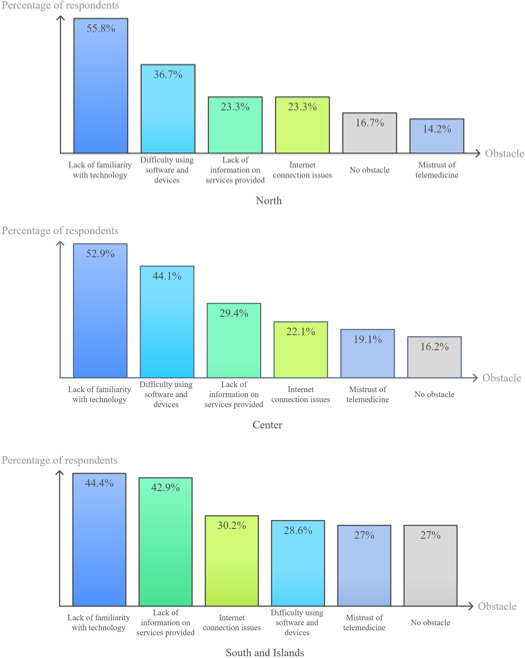
Barriers to patient uptake of telemedicine tools by geographical breakdown: north, center, and south and islands.

## Discussion

### Principal Findings

The response rate was in line with other published surveys, although not very high, and almost 80% of the centers reported telemedicine services in cardiology, most frequently telemonitoring, telereferrals, and medical teleconsultations. Heart failure was the disease for which these services were most frequently used. The possibility of reimbursement was present in less than one-half the cases; being guided by deliberations or operational protocols emerged as the situation for one-half the cases. The devices used in most facilities were medical devices, but health staff was rarely dedicated to these activities, trained in just over 55% of these activities. In addition, only 20% of staff had the ability to collect data. In only one-quarter of cases, patients and caregivers were not adequately informed.

The major reported concerns included a lack of dedicated staff, organizational complexity, and a lack of technological endowments available in the structure. For patients, the following were concerns: a lack of familiarity with technology, difficulties using software and devices, a lack of information, and a poor home internet connection.

These survey results warrant several considerations. The first concerns the response rate (56.7%), which, although not optimal, allowed the information of interest to be obtained from a considerable number of centers. Connection, cooperation, and dialogue between institutional organizations, which monitor and carry out research, and operational structures remain fundamental aspects to understand the critical points and solve them to build paths adapted to real-world needs and achieve results.

The most widespread telemedicine services and diseases treated through these tools would suggest that telemedicine could provide better home care, especially for chronic cases. This would allow early control of signs of instability or poor adherence to therapy, facilitating interception at the initial stages of clinical deterioration and a possible timely change in therapeutic strategy when the patient is still asymptomatic, before the onset of the acute event. However, the data on the effectiveness and impact of such interventions available in the literature are still controversial.

A systematic review conducted in 2022 analyzed 72 studies with more than 127,000 participants, showing that remote monitoring and consultations for patients with heart failure were associated with a reduced risk of cardiovascular mortality and hospitalization [14]. However, a review conducted in 2021 concluded that the pooled effect estimate of telemedicine interventions on all‐cause hospitalizations and all‐cause deaths in patients with recently decompensated heart failure and without implanted devices was neutral [15]. Similarly, another systematic review in the same year demonstrated that most telemonitoring programs do not show clear effects on health care utilization measures, except for an increase in nonemergency outpatient department visits [16].

Furthermore, there is a clear demand for greater professional interaction, which could accelerate the diagnostic and therapeutic process. Articles on teleconsultations published in the last 10 years show that there are positive impacts of teleconsultation such as improving patient management, but there are still gaps that need to be addressed [17].

There are still significant unresolved issues that limit the spread of telemedicine services, not only in the cardiology field but also regarding the reimbursement of telemedicine services, for example. At the international level, a qualitative study in the United States established that staff perceive current telehealth reimbursement policies as a factor that exacerbates inequities to accessing care. These findings indicate that, although telehealth brings new opportunities to advance patient-centered care, there are serious challenges on the path toward equitable care because telehealth is not yet integrated into the payment system in a sustainable way, and this is a factor exacerbating the lack of staff [18]. In Europe, a paper published in April 2025 [19] presented a comparative analysis of remote patient monitoring in 7 European countries (France, Germany, Italy, the Netherlands, Poland, Spain, and the United Kingdom): Germany introduced specific reimbursement codes for remote monitoring of patients with heart failure as early as 2020; the Netherlands has a relatively more advanced and flexible refund system, with dedicated codes for different digital performances; and in the United Kingdom, reimbursement is relatively more developed, especially in regions with more advanced health systems. In France, reimbursement for remote monitoring is still very limited and partial; in Poland, it is very limited; and in Spain, it is still poorly structured and often supported by pilot projects or temporary funds. Finally, as shown, in Italy, reimbursement is fragmented at the regional level, with some regions further ahead and others still in the embryonic stage. The inadequacy of traditional reimbursement models and the need for sustainable, evidence-based methods are highlighted as necessary for real digital transformation, as well as to obtain dedicated staff, which represents a major obstacle to development [20].

Additional obstacles are the resulting organizational complexity, also linked to the lack of appropriate organizational models supported by the presence of procedures and operational protocols that address and protect health personnel and patients [19]. Last but not least, the lack of adequate technological equipment remains a critical issue.

The centrality of the patient is a fundamental aspect. The data collected suggest that properly informing patients about these new services and methodologies is a need. This is fundamental to achieving good therapeutic adherence, increasing patient empowerment and engagement, and helping the patient and caregiver understand how much it can improve quality of life and therefore increase therapeutic continuity and avoid possible related risks [21-23]. Despite some geographical variability, the relatively low percentage of mistrust toward these new services (19% nationally, 14% in the north, 16% in the center, and 27% in the south) suggests that much was invested in recent times for patient centrality and providing information to patients and caregivers, probably obtaining good outcomes. The results show that 25% of patients are still not properly informed; it is necessary to supplement these data with evidence that some of the services provided (telereporting of ECGs, for example, or certain instrumental examinations) do not require detailed information to the patient on the functioning of the service itself. Unfortunately, it was not possible to perform a subanalysis to check in detail whether this percentage of “inadequate” information was related to these services, since the question relating to patient information was related to the telecardiology service in general and, therefore, to all services. Apart from this possible correlation, this lack of information provided to the patient justifies the main obstacles identified: the low familiarity with and difficulty using the essential technologies and the lack of information on the services provided.

### Limitations

The survey had some limitations regarding planning and dissemination. Since it was not possible to disseminate the survey through regional health departments, we contacted the facilities directly as described in the Methods section; thus, there is the possibility that we failed to contact some structures. In addition, considering the 11 questions that included a text description field, 7 of them asked the respondents to specify the cardiovascular diseases treated using telemedicine services. Due to the variety and dispersion of responses, it was difficult to elaborate answers to those questions; therefore, we decided to limit the analysis only to major cardiovascular pathologies, without providing further details. A final consideration concerns the remote referral: This service was investigated only in relation to instrumental examinations (eg, ECG, echocardiogram, monitoring of heart rhythm and blood pressure) without considering other services, televisits, and teleconsultation for which telereferring is provided.

Additionally, we cannot exclude that facilities using telemedicine services were more likely to participate in the survey than those without a telemedicine service. Finally, as this was a survey-based study, answers to some questions may have been affected by inaccuracies due to self-report bias or interpretation of the questions. Fortunately, the percentage of this event was very low.

### Conclusions

There are still organizational and clinical limitations in the application of telemedicine in cardiology, one of the first fields to use these services in real-life patient management; telemedicine in cardiology is not yet an integral part of medical practice. In fact, it appears that the spread of its use in Italy is still limited because of some structural and organizational barriers. Mainly, there is a lack of dedicated staff to manage and implement telemedicine services; sometimes, there are no clear rules and regulations on the uniform and safe adoption of these technologies at the national level or information about them. Finally, despite their wide availability, technologies are usually not well integrated into care pathways. These results confirm that the true challenge of telecardiology is the integration of available technology with precise, concrete, and simplified organizational models. As a tool, technology is fundamental only if it is accessible and adequate, and it must be integrated with new paths built according to the needs of the territory, patients, and health personnel. Fundamental aspects are reporting procedures and operating protocols to establish who does what and how and the availability of dedicated, adequately trained personnel who are able to inform patients and caregivers.

Such research could, in the future, help the implementation of a national plan for telemedicine services in cardiology and help create a telemedicine network to support continuous improvement of national and regional strategies.

## Supplementary material

10.2196/73747 Multimedia Appendix 1 Microsoft Forms questionnaire.

10.2196/73747 Multimedia Appendix 2 Additional information about the hospitals, centers, personnel, and devices.

10.2196/73747 Multimedia Appendix 3 List of health facilities who participated in the survey.

## Abbreviations

AIAC: Italian Association of Arrhythmology and Cardiostimulation
ANMCO: National Association of Doctors and Hospital Cardiologists
CHERRIES: Checklist for Reporting Results of Internet E-Surveys
CIED: cardiac implantable electronic device
ECG: electrocardiogram
ICT: information and communication technology
ISS: Italian National Institute of Health
OU: operative unit
TQoCT: Telehealth Quality of Care Tool
WHO: World Health Organization

## References

[1] (2010). Telemedicine: opportunities and developments in member states. report on the second global survey on eHealth. World Health Organization.

[2] (2018). Mapping of telemedicine experiences on the territory in 2018 [Website in Italian]. Ministry of Health.

[3] Gabbrielli F, Bertinato L, De Filippis G, Bonomini M, Cipolla M (2020). Interim indications for telemedicine care services during the COVID-19 health emergency. Report ISS COVID-19, no. 12/2020 [Website in Italian]. Italian National Institute of Health.

[4] (2020). Permanent conference for the relations between the state, the regions and the autonomous provinces of Trento and Bolzano. Agreement, pursuant to article 4, paragraph 1, of the legislative decree 28 August 1997, n. 281, between the government, the regions and the autonomous provinces of Trento and Bolzano, the provinces, the municipalities and the mountain communities, on the document "National indications for the provision of services in telemedicine" [Website in Italian]. Presidency of the Council of Ministers.

[5] Gabbrielli F, Gruppo di Consensus Nazionale sulla Telecardiologia (2023). Documento di consensus nazionale sullatelemedicina per le patologie cardiovascolari: indicazioni per la teleriabilitazione e il telemonitoraggio (Rapporti ISTISAN 23/21) [Report in Italian]. https://www.iss.it/documents/20126/9340234/23-21+web.pdf/edb447f0-19ba-71df-59c8-3db1ff88e696?t=1711719777176.

[6] Mahalwar G, Kumar A, Kalra A (2023). Virtual cardiology: past, present, future directions, and considerations. Curr Cardiovasc Risk Rep.

[7] Maines M, Palmisano P, Del Greco M (2021). Impact of COVID-19 pandemic on remote monitoring of cardiac implantable electronic devices in Italy: results of a survey promoted by AIAC (Italian Association of Arrhythmology and Cardiac Pacing). J Clin Med.

[8] Health Wellbeing and Resilience Observatory (2023). Connecting the dots: towards a national plan of health. Fondazione per la Ricerca Economica e Sociale ETS.

[9] Oliva F, Di Pasquale G, Lucci D (2024). 8th census of cardiology centers in Italy. Italian Association of Hospital Cardiologists (ANMCO). Year 2022. G Ital Cardiol (Rome).

[10] Elawady A, Khalil A, Assaf O, Toure S, Cassidy C (2020). Telemedicine during COVID-19: a survey of health care professionals’ perceptions. Monaldi Arch Chest Dis.

[11] Muehlensiepen F, Hoffmann MJ, Nübel J (2024). Acceptance of telemedicine by specialists and general practitioners in cardiology care: cross-sectional survey study. JMIR Form Res.

[12] Eysenbach G (2004). Improving the quality of web surveys: the Checklist for Reporting Results of Internet E-Surveys (CHERRIES). J Med Internet Res.

[13] (2024). Telehealth quality of care tool. World Health Organization.

[14] Kuan PX, Chan WK, Fern Ying DK (2022). Efficacy of telemedicine for the management of cardiovascular disease: a systematic review and meta-analysis. Lancet Digit Health.

[15] Drews TEI, Laukkanen J, Nieminen T (2021). Non-invasive home telemonitoring in patients with decompensated heart failure: a systematic review and meta-analysis. ESC Heart Fail.

[16] Auener SL, Remers TEP, van Dulmen SA, Westert GP, Kool RB, Jeurissen PPT (2021). The effect of noninvasive telemonitoring for chronic heart failure on health care utilization: systematic review. J Med Internet Res.

[17] Deldar K, Bahaadinbeigy K, Tara SM (2016). Teleconsultation and clinical decision making: a systematic review. Acta Inform Med.

[18] Porteny T, Brophy SA, Burroughs E (2025). Experiences of telehealth reimbursement policies in federally qualified health centers. JAMA Netw Open.

[19] Helms TM, Boriani G, Brunner-La Rocca HP (2025). The present and future of cardiological telemonitoring in Europe: a statement from seven European countries. Herzschr Elektrophys.

[20] Connolly J, Martin SS, Joynt Maddox KE, Maddox TM (2025). Reimbursement transformation Is essential for digital health adoption in cardiovascular care. JAMA Cardiol.

[21] Kruse CS, Krowski N, Rodriguez B, Tran L, Vela J, Brooks M (2017). Telehealth and patient satisfaction: a systematic review and narrative analysis. BMJ Open.

[22] Nielsen MK, Johannessen H (2019). Patient empowerment and involvement in telemedicine. J Nurs Educ Pract.

[23] Kruse C, Fohn J, Wilson N, Nunez Patlan E, Zipp S, Mileski M (2020). Utilization barriers and medical outcomes commensurate with the use of telehealth among older adults: systematic review. JMIR Med Inform.

