# Supplementary material for: Telecardiology Activities in Hospital and University Cardiology Facilities in Italy: Survey Study
Source: JMIR Cardio. 2025 Dec 5;9:e73747. doi: 10.2196/73747 (PMC12680089; doi:10.2196/73747)
Supplement: Multimedia Appendix 1 [file cardio-v9-e73747-s001.docx]

**Supplementary material**

Microsoft Forms Questionnaire.


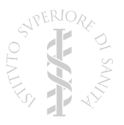
 **Survey on Telecardiology activities in Hospital and University Cardiology Facilities in Italy**

Dear Participant,

We would like to inform you that this initiative aims to carry out a survey on telemedicine in cardiology, to allow a photograph of the current state on national territory. The survey proposes, in detail, to assess the real territorial distribution of the use of telemedicine services in Cardiology, identifying the kind of telecardiology activities, design and delivery modalities, volumes and evaluation of users of services and, finally, to identify the obstacles to development, both in terms of health structure and in relation to the patients.

The objective is to make the survey as widespread as possible, in order to ensure a reliable and useful national photograph the future national design of telemedicine services in Cardiology and their actual functioning.

The data you provide will be used for the purpose of carrying out a survey within the Department of Diseases Cardiovascular, Endocrine-Metabolic and Aging of the Italian National Institute of Health. At the end of the census, the list of centres and the results of the survey will be published on the website of the Italian National Institute of Health and made available and searchable without registration.

By completing and sending this questionnaire, you express your consent to the processing of data in the manner and for the purposes described.

*Indicates a mandatory application.

**Details of the structure**

1. Region where the facility is located *

2. Province in which the facility is located *

3. City where the facility is located *

4. Structure of membership *

- Hospital directly managed by the local health company
- Hospital Company
- University Hospital Company
- University Hospital
- Other

5. Structure name*

6. Willingness to make available in the future the description of its Telemedicine projects in cardiology

- Yes
- No

**Telemedicine initiatives**

7. Has the facility ever taken Telemedicine initiatives? *

- Yes
- No

**Telemedicine activities – Typology**

8. Is there a televisit service (defined as a medical act where the professional interacts at a real time with the patient, also with the support of a care-giver)? *

- Yes
- No

9. Which pathology is addressed by the televisit service? *

- Therapeutic plans in the field of cardiovascular diseases
- High blood pressure
- Heart failure
- Ischemic heart disease
- Atrial fibrillation
- Syncope
- Arrhythmic disorders (to be specified in the next question)
- Remote control of implantable cardiac devices
- Valvular heart disease
- Rare cardiological diseases (to be specified in the next question)
- Congenital heart disease

10. If in the previous question it was indicated that the televisit service covers rare cardiological diseases and/or arrhythmias diseases, indicate in detail which pathologies

11. When did the televisit service start? Indicate the date. If the exact date is not available, indicate the first day of the month/year in which the service started. Example: if the service was started in January 2023, enter 01/01/2023

12. Is there a medical teleconsultation service (defined as a medical act where the professional interacts at a distance with one or more doctors to talk, including by video call, about the clinical situation of a patient)? *

- Yes
- No

13. The medical teleconsultation takes place with the following facilities *

- Clinical departments of the same hospital
- Other regional hospitals
- Other national hospitals or referral centres for pathology
- Private or private/contracted facilities
- General practitioner/paediatrician
- Territorial cardiology clinics
- Territorial non-cardiological specialist

14. Is the facility a hub centre, spoke or both? *

- Hub
- Spoke
- Both

15. Which pathology is the medical teleconsultation service intended for? *

- High blood pressure
- Heart failure
- Ischemic heart disease
- Atrial fibrillation
- Syncope
- Arrhythmic disorders (to be specified in the next question)
- Remote control of implantable cardiac devices
- Valvular heart disease
- Rare cardiological diseases (to be specified in the next question)
- Congenital heart disease
- Non-cardiological conditions (specify in the next question)

16. If answered that the medical teleconsultation service covers arrhythmic diseases and/or rare cardiological diseases and/or non-cardiological pathologies, indicate in detail such as arrhythmic pathologies, rare cardiological diseases and/or non-cardiological

17. When did the medical teleconsultation service start? Indicate the date. If the exact date is not available, indicate the first day of the month/year in which the service has been started. Example: if the service was started in January 2023, enter 01/01/2023

18. Is there is a health professionals teleconsultation service (not necessarily medical activity but still specific to the health professions, which takes place at a distance and is performed by two or more people with different responsibilities in relation to the specific case)?*

- Yes
- No

19. Who provides the health professionals teleconsultation service? *

- Nurses
- Cardio-circulatory pathophysiology technicians
- Bioengineers
- Psychologists
- Physiotherapists
- Other

20. What pathology is the health professionals’ teleconsultation service intended for? *

- High blood pressure
- Heart failure
- Ischemic heart disease
- Atrial fibrillation
- Syncope
- Arrhythmic disorders (to be specified in the next question)
- Remote control of implantable cardiac devices
- Valvular heart disease
- Rare cardiological diseases (to be specified in the next question)
- Congenital heart disease
- Non-cardiological conditions (to be specified in the next application)

21. If answered that the teleconsultation service covers arrhythmic and/or rare cardiological and/or non-cardiological diseases, give details of arrhythmic diseases, rare cardiological diseases and/or non-cardiological diseases

22. When did health professionals’ teleconsultation service start? Indicate the date. If the exact date is not available, indicate the first day of the month/year in which the service is has been started. Example: if the service was started in January 2023, enter 01/01/2023

23. Is there a telemonitoring service (defined as an act that allows the detection and transmission to distance of vital and clinical parameters continuously, by means of interacting sensors with the patient)? *

- Yes
- No

24. Who provides the telemonitoring service? *

- Nurses
- Cardio-circulatory pathophysiology technicians
- Medici

25. Which pathology is the telemonitoring service intended for? *

- High blood pressure
- Heart failure
- Ischemic heart disease
- Atrial fibrillation
- Syncope
- Arrhythmic disorders (to be specified in the next question)
- Remote control of implantable cardiac devices
- Valvular heart disease
- Rare cardiological diseases (to be specified in the next question)
- Congenital heart disease
- Non-cardiological conditions (to be specified in the next application)

26. If answered that the telemonitoring service covers arrhythmic and/or rare cardiological and/or non-cardiological diseases, give details of arrhythmic diseases, rare cardiological diseases and/or non-cardiological diseases

27. When did the telemonitoring service start? Please indicate the date. If the exact date is not available, indicate the first day of the month/year in which the service was started. Example: if the service started in January 2023, enter 01/01/2023

28. Is there a tele referral service (defined as a report issued by the doctor who has submitted a patient to a clinical or instrumental examination whose content is typical of referrals and it is written and transmitted by means of digital systems telecommunications)? *

- Yes
- No

29. What is the tele referral service for? *

- Emergency ECG (network 118)
- Intra-hospital ECG
- ECG hospital-territory
- Holter ECG monitoring 24 hours
- ECG monitoring through other devices (specify in the next question)
- Outpatient monitoring of blood pressure
- Echocardiogram
- Remote control of implantable cardiac devices

30. If answered that the tele referral service is addressed to ECG monitoring through other devices in the previous question, please indicate in detail which device

31. When did the tele referral service start? Please indicate the date. If the precise date is not available, indicate the first day of the month/year in which the service was started. Example: if the service started in January 2023, enter 01/01/2023

32. Is there is a tele rehabilitation service (defined as the remote provision of services and intended to enable, restore, improve or otherwise maintain psycho-physical functioning of people of all ages, with disabilities or disorders, congenital or acquired, transient or permanent, or at risk of developing them. It is a health activity relevant to professionals’ health)? *

- Yes
- No

33. What is the tele rehabilitation service for? *

- Heart failure
- Ischemic heart disease
- Arrhythmia
- Valvular heart disease
- Rare diseases in cardiology
- Congenital heart disease

34. When did the tele rehabilitation service start? Please indicate the date. If the exact date is not available, indicate the first day of the month/year in which the service was started. Example: if the service started in January 2023, enter 01/01/2023

**Telemedicine activities - Governance, security and accessibility for healthcare professionals**

35. Is there a provision is made for a charging system for telemedicine services in the region of the facility? *

- Yes
- No

36. Which services are charged? *

- Televisit
- Medical teleconsultation
- Health professionals’ teleconsultation
- Telemonitoring
- Remote control (CIED)
- Tele rehabilitation
- Other

37. Has the facility made available business process or delivery protocols, according to national guidelines and documents? *

- Yes, business deliberations with procedures
- Yes, shared operating protocols
- Yes, informed consent models for users
- No
- Other

38. Has the facility or business unit provided training for healthcare staff when telemedicine services are started? *

- Yes
- No

39. How are telemedicine services delivered? *

- Regional Platform
- Dedicated web platform of the facility (website)
- Non-dedicated platform via web (Facebook, Twitter, etc...)
- Dedicated platform via app (mobile app, in case specify which app it is in the next question)
- Non-dedicated platform via app (WhatsApp, Telegram, Messenger, etc...)
- Encrypted e-mail address
- Unencrypted e-mail address
- Electronic health record
- Other

40. If the previous question indicated that telemedicine services are delivered via a dedicated app platform, please indicate in detail which apps are used

41. Is the use of devices planned? *

- Yes
- No

42. If yes, which devices are used?

- Device for frequency control
- Blood pressure monitoring device
- ECG
- Saturation meter
- Libra
- Ultrasound
- Impedance meter
- Spirometer
- Glucometer
- CIED
- Other

43. Are the devices used configured as medical devices? *

- Yes
- No
- I do not know

44. Is there a telemedicine centre at the facility with dedicated staff? *

- Yes
- No

45. Is there any plan to collect data in a dedicated database? *

- Yes
- No

46. Specify which database is used *

**Telemedicine activities - patient volumes and centrality**

47. How many patients are followed on average in a year through telemedicine services? * The value should be a number

48. Is there an assessment of patient satisfaction with telemedicine services? *

- Yes
- No

49. By which means? *

- Questionnaires
- Interviews
- Other

50. Does the facility or unit provide information tools for patients and their caregivers about telemedicine services and the use of necessary technology? *

- Yes, through in presence events
- Yes, by means of information brochures
- Yes, by means of material in digital format on the website
- Yes, the information is in a personalized form at the start of the telemedicine path
- No

**Telemedicine activities - Obstacles to development**

51. What obstacles have been found to the adoption of telemedicine tools by health care facility?

- Cost in economic terms
- Complexity in organisational terms
- Lack of technological equipment in the structure
- Lack of adequate infrastructure and Internet connectivity
- Data fragmentation and non-interoperability of systems
- Lack of legislation
- Complexity in the application of specific legislation
- Complexity in the application of GDPR
- Mistrust and lack of confidence in telemedicine by the health establishment
- Lack of staff willingness or cooperation
- Lack of dedicated staff
- Internal bureaucratic procedures inappropriate to the theme
- Poor digital training of staff
- No obstacles

52. What obstacles have been encountered in the adoption of telemedicine tools for patients? *

- Lack of information on telemedicine services
- Difficulty in using the necessary software and devices
- Lack of familiarity with information technology
- Internet connections to patient’s home are not efficient and reliable
- Mistrust of Telemedicine
- No obstacles.
