# Supplementary material for: Telecardiology Activities in Hospital and University Cardiology Facilities in Italy: Survey Study
Source: JMIR Cardio. 2025 Dec 5;9:e73747. doi: 10.2196/73747 (PMC12680089; doi:10.2196/73747)
Supplement: Multimedia Appendix 2 [file cardio-v9-e73747-s002.docx]

**Supplementary material**

**Table S1**. Distribution of the type of hospital structure, at national level and by geographical area.

| **Geographical area** | **Local health authority Hospital** | **Independent Hospital** | **University Hospital** | **University Polyclinic** | **Other** | **Total** |
| --- | --- | --- | --- | --- | --- | --- |
|  | n (%) | n (%) | n (%) | n (%) | n (%) | n |
|  |  |  |  |  |  |  |
| North | 49 (40.8) | 41 (34.2) | 16 (13.3) | 2 (1.7) | 12 (10.0) | 120 |
|  |  |  |  |  |  |  |
| Centre | 38 (55.9) | 10 (14.7) | 5 (7.3) | 8 (11.8) | 7 (10.3) | 68 |
|  |  |  |  |  |  |  |
| South and islands | 36 (57.1) | 13 (20.6) | 12 (19.1) | 0 | 2 (3.2) | 63 |
|  |  |  |  |  |  |  |
| Total | 123 (49.0) | 64 (25.5) | 33 (13.1) | 10 (4.0) | 21 (8.4) | 251 |

**Table S2**. Type of centres (hub and spoke) for medical teleconsultation, reported by geographical area.

| **Type of structure** | **North** | **Centre** | **South and Islands** | **Total** |
| --- | --- | --- | --- | --- |
|  | **(n=52)** | **(n=28)** | **(n=13)** | **(n=93)** |
|  | n (%) | n (%) | n (%) | n (%) |
|  |  |  |  |  |
| Hub | 13 (25.0) | 8 (28.6) | 7 (53.8) | 28 (30.1) |
|  |  |  |  |  |
| Spoke | 22 (42.3) | 11 (39.3) | 4 (30.8) | 37 (39.8) |
|  |  |  |  |  |
| Both | 17 (32.7) | 9 (32.1) | 2 (15.4) | 28 (30.1) |

**Table S3**. Personnel performing telemonitoring services, at national level and by geographical area.

| **Professional role** | **North** | **Centre** | **South and Islands** | **Total** |
| --- | --- | --- | --- | --- |
|  | **(n=73)** | **(n=34)** | **(n=21)** | **(n=128)** |
|  | n (%) | n (%) | n (%) | n (%) |
|  |  |  |  |  |
| Nurses | 55 (75.3) | 14 (41.2) | 13 (61.9) | 82 (64.0) |
|  |  |  |  |  |
| Doctors | 51 (69.9) | 23 (67.6) | 16 (76.2) | 90 (70.3) |
|  |  |  |  |  |
| Cardio-circulatory pathophysiology technicians | 26 (35.6) | 15 (44.1) | 4 (19.0) | 45 (35.2) |

**Table S4**. Centres that use deliberations, procedures, protocols, informed consents, at national level and by geographical area.

| **Type of document in use** | **North** | **Centre** | **South and Islands** | **Total** |
| --- | --- | --- | --- | --- |
|  | **(n=57)** | **(n=26)** | **(n=17)** | **(n=101)** |
|  | n (%) | n (%) | n (%) | n (%) |
| Business deliberations with procedures | 28 (49.1) | 7 (26.9) | 8 (47.1) | 43 (42.6) |
|  |  |  |  |  |
| Informed consent models for users | 23 (40.3) | 10 (38.5) | 7 (41.2) | 40 (39.6) |
|  |  |  |  |  |
| Shared operational protocols | 36 (63.2) | 12 (46.1) | 5 (29.4) | 53 (52.5) |

Table S5. Device types used in telemedicine, at a national level and by geographical area.

| **Device types** | **Total** | **North** | **Centre** | **South and Islands** |
| --- | --- | --- | --- | --- |
|  | **(n=198)** | **(n=101)** | **(n=53)** | **(n=44)** |
|  | n (%) | n (%) | n (%) | n (%) |
|  |  |  |  |  |
| ^a^Physical activity, sleep | 1 (0.5) | 0 | 1 (1.9) | 0 |
|  |  |  |  |  |
| ^a^Weighing machine | 26 (13.1) | 12 (11.9) | 6 (11.3) | 8 (18.1) |
|  |  |  |  |  |
| ^b^CIED | 52 (26.3) | 26 (25.7) | 14 (26.4) | 12 (27.3) |
|  |  |  |  |  |
| ^a^Device for home transmission | 1 (0.5) | 0 | 0 | 1 (2.3) |
|  |  |  |  |  |
| ^a^Device for frequency control | 39 (19.7) | 15 (14.8) | 13 (24.5) | 11 (25.0) |
|  |  |  |  |  |
| ^a^Blood pressure monitoring device | 33 (16.7) | 14 (13.9) | 8 (15.1) | 11 (25.0) |
|  |  |  |  |  |
| ^a^Hemodynamic monitoring devices | 1 (0.5) | 1 (1) | 0 | 0 |
|  |  |  |  |  |
| ECG | 48 (24.2) | 18 (17.8) | 12 (22.6) | 18 (40.9) |
|  |  |  |  |  |
| Ultrasound | 2 (1.0) | 0 | 1 (1.9) | 1 (2.3) |
|  |  |  |  |  |
| Event recorder | 1 (0.5) | 0 | 1 (1.9) | 0 |
|  |  |  |  |  |
| ^a^Glucometer | 2 (1) | 0 | 0 | 2 (4.5) |
|  |  |  |  |  |
| ^a^Impedance meter | 5 (2.5) | 2 (2) | 2 (3.8) | 1 (2.3) |
|  |  |  |  |  |
| ^b^Loop recorder | 1 (0.5) | 1 (1) | 0 | 0 |
|  |  |  |  |  |
| ^b^PM monitoring | 1 (0.5) | 0 | 1 (1.9) | 0 |
|  |  |  |  |  |
| ^b^PM-ICD and Loop Recorder carriers | 1 (0.5) | 0 | 0 | 1 (2.3) |
|  |  |  |  |  |
| ^a^Pulse oximeter | 30 (15.1) | 15 (14.8) | 5 (9.4) | 10 (22.7) |
|  |  |  |  |  |
| ^a^Spirometer | 4 (2.0) | 3 (3.0) | 1 (1.9) | 0 |
|  |  |  |  |  |
| ^a^Temperature and respiratory rate | 1 (0.5) | 1 (1.0) | 0 | 0 |
|  |  |  |  |  |
| Other | 1 (0.5) | 1 (1.0) | 0 | 0 |

CIED: cardiac implantable electronic device; ECG: electrocardiogram; PM: pacemaker; ICD: implantable cardioverter defibrillator.

^a^Device for clinical parameters monitoring; ^b^Implantable device.
