# Supplementary material for: Telecardiology Activities in Hospital and University Cardiology Facilities in Italy: Survey Study
Source: JMIR Cardio. 2025 Dec 5;9:e73747. doi: 10.2196/73747 (PMC12680089; doi:10.2196/73747)
Supplement: Multimedia Appendix 3 [file cardio-v9-e73747-s003.docx]

**Supplementary material**

List of health facilities who participated in the survey**.**

Please note that the number of facilities does not correspond to the total number of responses received, as sometimes several operating units are located in the same hospital.

- Regional Hospital U. Parini of Aosta
- Santo Spirito Hospital of Casale Monferrato (AL)
- Presidio Civile Santi Antonio e Biagio of Alessandria
- Cardinal Massaia Hospital of Asti
- SS. Trinità Hospital of Fossano (CN)
- SS. Annunziata Hospital of Savigliano (CN)
- Regina Montis Regalis Hospital in Mondovi (CN)
- Ospedale Maggiore della Carità of Novara
- Santissima Trinità Hospital of Borgomanero (NO)
- Sant'Andrea Hospital in Vercelli
- SS. Pietro and Paolo Hospital of Borgosesia (VC)
- Ospedale degli Infermi of Ponderano (BI)
- Presidi Ospedalieri Castelli di Verbania and San Biagio of Domodossola (VCO)
- San Giuseppe Hospital in Oggebbio (VCO)
- Martini - Maria Vittoria Hospital of Turin
- Hospital-University of the City of Health and Science of Turin
- Mauritian Hospital in Turin
- San Giovanni Bosco Hospital in Turin
- Ospedale degli Infermi of Rivoli (TO)
- University Hospital San Luigi Gonzaga of Orbassano (TO)
- Hospital of Ivrea (TO)
- Hospital of Chivasso (TO)
- Civil Hospital Agnelli of Pinerolo (TO)
- Santa Croce Hospital of Moncalieri (TO)
- Policlinico San Martino of Genoa
- Hospital P.A. Micone in Sestri Ponente (GE)
- Lavagna Hospital (GE)
- Hospital of Rapallo (GE)
- Civil Hospital of Imperia
- San Paolo Hospital of Savona
- Presidio Ospedaliero Levante Ligure S. Andrea - Sarzana of La Spezia
- Polyclinic of Milan
- Niguarda Hospital in Milan
- San Carlo Hospital in Milan
- Luigi Sacco Hospital in Milan
- San Luca Hospital in Milan
- Hospital Gaetano Pini CTO Rehabilitation of Milan
- Hospital of Legnano (MI)
- Hospital of Cuggiono (MI)
- Hospital Uboldo of Cernusco on Naviglio (MI)
- Major Hospital of Lodi
- Civil Hospital of Codogno (LO)
- Circolo and Fondazione Macchi of Varese
- Galmarini Hospital of Tradate (VA)
- Hospital of Saronno (VA)
- Hospital of Lecco
- Merate Hospital (LC)
- Hospital of Seregno (MB)
- Hospital of Vimercate (MB)
- Hospital "SS. Trinità" of Romano (BG)
- Bolognini Hospital of Seriate (BG)
- Spedali Civili of Brescia
- Hospital of Gardone Val Trompia (BS)
- Chiari Hospital (BS)
- Hospital of Esine (BS)
- Hospital of Desenzano del Garda (BS)
- Ospedale Civile La Memoria of Gavardo (BS)
- San Matteo Polyclinic of Pavia
- Civil Hospital of Vigevano (PV)
- Civil Hospital of Voghera (PV)
- Major Hospital of Crema (CR)
- Santa Marta Hospital of Rivolta d'Adda (CR)
- Hospital of Borgo Mantovano (MN)
- Corrado Poma Hospital in Mantua
- Santa Maria del Carmine Hospital in Rovereto (TN)
- Hospital of Bolzano
- Hospital of Merano (BZ)
- Presidio Ospedaliero San Martino of Belluno
- Presidio Ospedaliero Santa Maria del Prato of Feltre (BL)
- Cà Foncello Hospital of Treviso
- Civil Hospital of Conegliano (TV)
- Hospital of Montebelluna (TV)
- Noale Hospital (VE)
- Civil Hospital SS Giovanni and Paolo of Venice
- Civil Hospital of San Donà di Piave and Portogruaro (VE)
- Veneto Cancer Institute of Padua
- Hospital of Camposampiero (PA)
- Hospital of Cittadella (PA)
- San Bortolo Hospital in Vicenza
- Hospital of Bassano (VI)
- Hospital of Asiago (VI)
- Cazzavillan Hospital of Arzignano (VI)
- Fracastoro Hospital of San Bonifacio (VE)
- Orlandi Hospital in Bussolengo and Magalini Hospital in Villafranca (VE)
- Sacro Cuore Don Calabria Hospital of Negrar di Valpollicella (VE)
- Presidio Ospedaliero Universitario Santa Maria della Misericordia in Udine
- Institute of Physical Medicine for Cardiological Rehabilitation of Gervasutta (UD)
- Giuliano Isontina University Hospital of Cattinara, Trieste
- Hospital of Gorizia and Monfalcone
- Hospital Santa Maria degli Angeli of Pordenone
- Hospital Guglielmo da Saliceto di Piacenza
- Castel San Giovanni Val Tidone Hospital (PC)
- Hospital of Fidenza (PR)
- Santa Maria di Borgo Val di Taro Civil Hospital (PR)
- St. Anna’s Hospital in Castelnovo ne' Monti (RE)
- Hospital of Sassuolo (MO)
- Santa Maria Bianca Hospital in Mirandola (MO)
- Sant'Orsola Hospital in Bologna
- Bologna Major Hospital
- Bellaria Hospital in Bologna
- Dossetti Hospital in Bazzano (BO)
- Bentivoglio Hospital (BO)
- SS. Annunziata Hospital of Cento (FE)
- Sant'Anna Archhospital in Ferrara
- Hospital M. Bufalini of Cesena
- Hospital G.B. Morgagni L. Pierantoni di Forlì
- Ospedale degli Infermi in Faenza (RA)
- Santa Maria delle Croci Hospital in Ravenna
- Civil Hospital of Lugo (RA)
- Santa Maria Nuova Hospital in Florence
- University Hospital Careggi of Florence
- Mugello New Hospital of Borgo San Lorenzo (FI)
- Santa Maria Annunziata Hospital in Bagno a Ripoli (FI)
- San Giuseppe Hospital of Empoli (FI)
- Santi Cosma e Damiano Hospital of Valdinievole-Pescia (PT)
- Santo Stefano Hospital di Prato (PT)
- Santa Croce Hospital of Castelnuovo Garfagnana (LU)
- San Luca Hospital di Lucca
- New Versilia Hospital of Lido di Camaiore (LU)
- Hospital of Cecina (LI)
- Apuan Hospital - NOA of Massa e Carrara
- Sant'Andrea Hospital of Massa e Carrara
- Felice Lotti Hospital in Pontedera (PI)
- University Hospital Le Scotte in Siena
- Campostaggia Hospital of Poggibonsi (SI)
- San Donato Hospital of Arezzo
- Valdarno La Gruccia Hospital of Montevarchi (AR)
- La Fratta Hospital of Cortona (AR)
- Misericordia Hospital of Grosseto
- Carlo Urbani Hospital in Jesi (AN)
- E. Profili Hospital of Fabriano (AN)
- Hospital of Macerata
- Santa Maria della Pietà Hospital of Camerino (MC)
- Hospital of Civitanova Marche (MC)
- City of Castello Hospital (PG)
- Hospital of Gubbio-Gualdo Tadino (PG)
- San Giovanni Battista Hospital in Foligno (PG)
- Media Valle del Tevere Hospital in Todi (PG)
- Santa Maria Hospital of Terni
- San Camillo de Lellis Hospital in Rieti
- San Paolo Hospital of Civitavecchia
- Santa Maria Goretti Hospital in Latina
- Santa Scolastica Hospital of Cassino (FR)
- Presidio Ospedaliero in Sora (FR)
- Hospital of Anzio (RM)
- Hospital of Frascati (RM)
- Hospital of Colleferro (RM)
- Tivoli Hospital (RM)
- Presidio Ospedaliero Santo Spirito in Sassia of Rome
- Presidio Ospedaliero San Filippo Neri of Rome
- Azienda Ospedaliero-Universitaria Sant'Andrea of Rome
- Sandro Pertini Hospital in Rome
- Sant'Eugenio Hospital in Rome
- G.B. Grassi Hospital in Rome
- Isola Tiberina Hospital in Rome
- Policlinico Casilino of Rome
- San Camillo Forlanini Hospital of Rome
- C.T.O. Andrea Alesini of Rome
- San Giovanni Addolorata Hospital of Rome
- A. Gemelli Foundation University Polyclinic of Rome
- San Raffaele Hospital in Rome
- INMI Lazzaro Spallanzani of Rome
- Dermopatic Institute of the Immaculate of Rome
- Cristo Re Hospital of Rome
- San Timoteo Hospital in Termoli (CB)
- Presidio Santa Maria Capua Vetere Hospital (CE)
- Hospital of Sessa Aurunca (CE)
- Sacro Cuore di Gesù Fatebenefratelli Hospital of Benevento
- S. Maria della Speranza Hospital of Battipaglia (SA)
- S. Maria Coronata of the Olmo Hospital in Cava de' Tirreni (SA)
- Luigi Vanvitelli University Hospital of Campania
- Antonio Cardarelli Hospital of Naples
- Pilgrims Hospital of Naples
- San Paolo Hospital in Naples
- San Giovanni Bosco Hospital in Naples
- Ospedale del Mare of Naples
- Anna Rizzoli Hospital of Ischia (NA)
- Giovanni XXIII Polyclinic in Bari
- San Paolo Hospital in Bari
- San Giacomo Hospital of Monopoli (BA)
- Santa Maria degli Angeli Hospital in Putignano (BA)
- M. Sarcone Hospital of Terlizzi (BA)
- Caduti in Guerra Hospital of Canosa di Puglia (BT)
- Di Summa - Perrino Presidio of Brindisi
- University Hospital Policlinico Riuniti in Foggia
- Teresa Masselli Mascia Hospital of San Severo (FG)
- San Giuseppe da Copertino Hospital in Copertino (LE)
- Veris Delli Ponti Hospital of Scorrano (LE)
- Vito Fazzi Hospital in Lecce
- Presidio Ospedaliero Centrale - SS. Annunziata of Taranto
- Regional Hospital of San Carlo in Potenza
- Hospital of Soverato (CZ)
- Hospital of Cosenza
- Hospital of Castrovillari (CS)
- Hospital of Corigliano-Rossano (CS)
- Azienda Ospedaliera Bianchi Melacrino Morelli of Reggio Calabria
- Civil Hospital of Locri (RC)
- Licata Hospital Presidium (AG)
- Vittorio Emanuele Hospital of Gela (CL)
- San Salvatore Hospital in Paternò-Biancavilla Bronte (CT)
- University Hospital Policlinico G. Rodolico - San Marco in Catania
- Hospital for the Emergency Cannizzaro of Catania
- Garibaldi-Centro Hospital of Catania
- Garibaldi-Nesima Hospital of Catania
- Umberto I Hospital of Enna
- Nicosia Hospital (EN)
- University Hospital of Messina
- Hospital Giuseppe Fogliani of Milazzo (ME)
- Presidio Ospedaliero G.F. Ingrassia of Palermo
- Villa Sofia Hospital of Palermo
- Giovanni Paolo II Hospital in Ragusa
- Ospedale Maggiore of Modica (RG)
- E. Muscatello Hospital in Augusta (SR)
- S.Antonio Abate Hospital/Vittorio Emanuele II Hospital of Trapani-Castelvetrano
- Paolo Borsellino Hospital in Marsala (TP)
- San Vito e Santo Spirito Hospital of Alcamo (TP)
- Duilio Casula University Hospital of Cagliari
- ARNAS "G. Brotzu" San Michele Hospital in Cagliari
- Santissima Trinità Hospital of Cagliari
- SS. Annunziata Hospital of Sassari
- Giovanni Paolo II Hospital of Olbia (SS)
- San Francesco Hospital of Nuoro
- Nostra Signora della Mercede Hospital in Lanusei (NU)
- San Martino Hospital in Oristano
- Nostra Signora di Bonaria Hospital in San Gavino Monreale (SU).
